# Mechanisms of Normalisation of Bone Metabolism during Recovery from Hyperthyroidism: Potential Role for Sclerostin and Parathyroid Hormone

**DOI:** 10.1155/2015/948384

**Published:** 2015-08-23

**Authors:** Elżbieta Skowrońska-Jóźwiak, Krzysztof C. Lewandowski, Zbigniew Adamczewski, Kinga Krawczyk-Rusiecka, Andrzej Lewiński

**Affiliations:** Department of Endocrinology and Metabolic Diseases, Medical University of Lodz, 281/289 Rzgowska Street, 90-338 Lodz, Poland

## Abstract

Sclerostin, a protein expressed by osteocytes, is a negative regulator of bone formation. The aim of the study was to investigate the relationship between parathyroid hormone (PTH) and markers of bone metabolism and changes of sclerostin concentrations before and after treatment of hyperthyroidism. *Patients and Methods*. The study involved 33 patients (26 women), age (mean ± SD) 48 ± 15 years, with hyperthyroidism. Serum sclerostin, PTH, calcium, and bone markers [osteocalcin (OC) and collagen type I cross-linked C-telopeptide I (CTX)] were measured at diagnosis of hyperthyroidism and after treatment with thiamazole. *Results*. After treatment of hyperthyroidism a significant decrease in free T_3_ (FT_3_) and free T_4_ (FT_4_) concentrations was accompanied by marked decrease of serum sclerostin (from 43.7 ± 29.3 to 28.1 ± 18.4 pmol/L; *p* < 0.001), OC (from 35.6 ± 22.0 to 27.0 ± 14.3 ng/mL; *p* < 0.001), and CTX (from 0.49 ± 0.35 to 0.35 ± 0.23 ng/dL; *p* < 0.005), accompanied by an increase of PTH (from 29.3 ± 14.9 to 39.8 ± 19.8; *p* < 0.001). During hyperthyroidism there was a positive correlation between sclerostin and CTX (*r*_*s*_ = 0.41, *p* < 0.05) and between OC and thyroid hormones (with FT_3_ *r*_*s*_ = 0.42, with FT_4_ *r*_*s*_ = 0.45, *p* < 0.05). *Conclusions*. Successful treatment of hyperthyroidism results in a significant decrease in serum sclerostin and bone markers concentrations, accompanied by an increase of PTH.

## 1. Introduction

Sclerostin is a product of a SOST gene, expressed mainly by osteocytes. This protein is crucial for inhibiting bone formation, by decreasing proliferation and differentiation of osteoblasts, reducing apoptosis of mature osteoblasts, thus leading to higher bone formation and increased bone density [1]. Sclerostin inhibits canonical Wnt/*β*-catenin signalling by binding to LRP-5 and LRP-6 [2]. Sclerostin levels are inhibited by mechanical loading and parathormone (PTH) [1, 3] and stimulated by glucocorticoids and calcitriol [1]. Other determinants of sclerostin concentrations include BMI, sex, age, and several hormones, such as follicle stimulating hormone (FSH) and estradiol (E2) [3, 4]. Sclerostin deficiency has been implicated in the pathogenesis of rare bone dysplasias, that is, sclerosteosis and van Buchem disease, where mechanical strength of bone is extremely large [1]. This has led to the idea of implementing antisclerostin interventions in therapy of osteoporosis. Monoclonal antibodies to sclerostin, including romosozumab, blosozumab, and BPS804, were demonstrated to increase bone mineral density and to stimulate bone formation together with reducing bone resorption [5].

We have previously shown a decrease of sclerostin levels during successful treatment of hyperthyroidism in a preliminary study, involving 15 patients [6]. In order to further elucidate the mechanism of that phenomenon we measured PTH, calcium, and markers of bone metabolism in blood samples taken from the patients before and during treatment of hyperthyroidism.

## 2. Patients and Methods

The study involved 33 patients (7 men), age 48 ± 15 years, BMI 24.2 ± 3.5 kg/m^2^, with hyperthyroidism due to Graves' disease (*n* = 15) or toxic multinodular goitre hospitalized in the Department of Endocrinology and Metabolic Diseases. Patients had no history of bone diseases and therapy affecting bone metabolism. Patients signed informed consent. The study had been accepted by Bioethics Committee of Medical University of Lodz. Characteristics of investigated population before and after treatment are shown in Table 1. All patients received a thyrostatic drug, thiamazole, and beta-blocker, propranolol.

Serum sclerostin was measured by a quantitative sandwich ELISA by Biomedica (Vienna, Austria) at diagnosis of hyperthyroidism and after 6–10 weeks of treatment with thiamazole. The intra-assay CV is 5%, and the interassay CV is 3–6%.

Thyroid-stimulating hormone (TSH), free T_3_ (FT_3_), free T_4_ (FT_4_), PTH, osteocalcin (OC), marker of bone formation, and collagen type I cross-linked C-telopeptide I (CTX), marker of bone resorption, were determined by commercially available electrochemiluminescence immunoassays (ECLIA Cobas e601, Roche). The intra-assay and interassay CVs were less than 4.0%. Calcium was measured by colorimetric method (Vitros 5.1).

The data were analysed by means of simple descriptive statistics of location and dispersion. A comparison of distributions of selected parameters before and during treatment was assessed by Wilcoxon's matched pairs test. Association between variables was evaluated with use of Spearman's correlation coefficient. Analysis of relationship between sclerostin and demographic data (BMI and age) was done assessed by method of multiple correlation. In all analyses, statistical significance was considered achieved for a value of *p* ≤ 0.05. Calculations were performed by means of Statistica 10.0 software.

## 3. Results

After treatment of hyperthyroidism, a significant decrease in FT_3_ from 10.2 ± 5.4 pg/mL to 3.2 ± 1.0 pg/mL (*p* < 0.000001) and FT_4_ concentrations from 4.03 ± 2.33 ng/mL to 1.10 ± 0.82 ng/mL, respectively (*p* < 0.006), was accompanied by a marked decrease of serum sclerostin levels from 43.7 ± 29.2 to 28.1 ± 18.4 pmol/L (*p* = 0.000001). There was a simultaneous decrease of OC from 35.6 ± 22.0 to 27.0 ± 14.3 ng/mL, *p* = 0.00004, and CTX from 0.49 ± 0.35 to 0.35 ± 0.23 ng/dL, *p* = 0.0016. In contrast, PTH concentrations increased from 29.3 ± 14.9 pg/mL to 39.8 ± 19.8 pg/mL, *p* = 0.0005 (Table 2).

**Figure 1:**
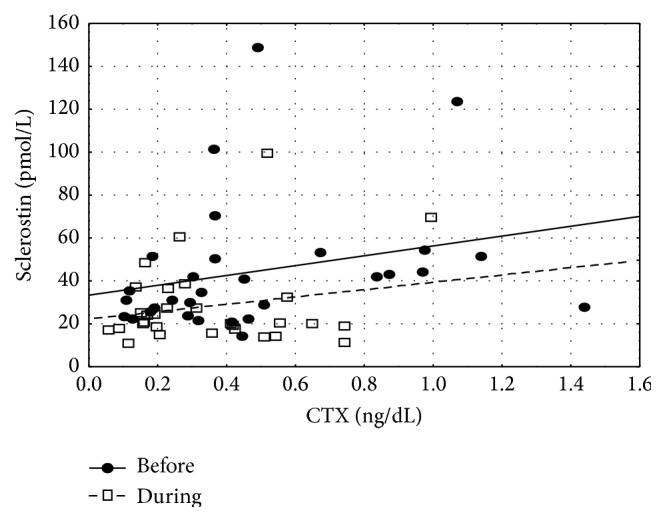
Scatterplot for sclerostin and CTX (before hyperthyroidism treatment *r*_*s*_ = 0.41, *p* < 0.05).

During thyrotoxic phase sclerostin correlated positively with CTX (*r*_*s*_ = 0.41, *p* < 0.05) (Figure 1).

Sclerostin concentrations did not correlate with PTH levels (*r* = 0.18, *p* = NS). In order to assess whether individuals with maximal changes in sclerostin also had maximal changes in PTH concentrations, we analysed individual changes of sclerostin concentrations before and after treatment of thyrotoxicosis (i.e., Δsclerostin) and correlated these data with individual changes of PTH levels before and after treatment of thyrotoxicosis (i.e., ΔPTH). Here again we did not observe any significant correlation between Δsclerostin and ΔPTH (*r* = −0.10, *p* = NS).

**Figure 2:**
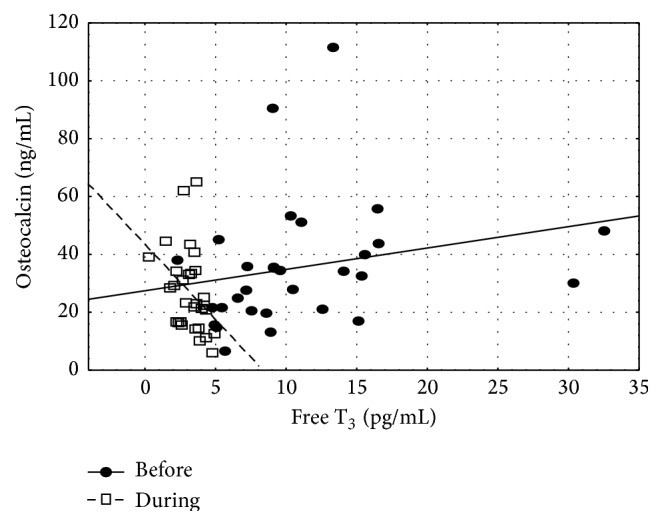
Scatterplot for osteocalcin and free T_3_, before and during hyperthyroidism treatment. Before treatment *r*_*s*_ = 0.42, after treatment *r*_*s*_ = −0.45, *p* < 0.05.

Before treatment OC correlated positively with thyroid hormones, both FT_4_ (*r*_*s*_ = 0.45, *p* < 0.05) and FT_3_ (*r*_*s*_ = 0.42, *p* < 0.05), but after treatment we observed negative correlation between OC and FT_3_ (*r*_*s*_ = −0.43, *p* < 0.05) (Figure 2).

**Figure 3:**
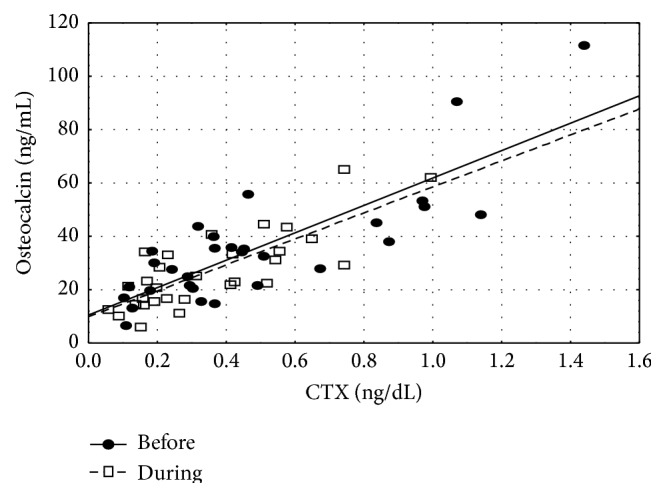
Scatterplot for osteocalcin and CTX, before and during hyperthyroidism treatment (*r*_*s*_ = 0.76, both before and after treatment, *p* < 0.01).

Moreover, before therapy we demonstrated a strong positive correlation between OC and CTX (*r*_*s*_ = 0.76, *p* < 0.01) (Figure 3) and negative correlation with age (*r*_*s*_ = −0.43, *p* < 0.05).

Tables 3 and 4 summarize correlations before and during treatment, respectively.

## 4. Discussion

We demonstrated that restoration of an euthyroid state after treatment of thyrotoxicosis is associated with a significant decrease of sclerostin, osteocalcin, and CTX serum concentrations coinciding with an increase of PTH concentrations.

The influence of thyroid hormones on bone metabolism is multifaceted and depends on age. During growth thyroid hormones have predominantly anabolic actions in bone, while in adulthood they predominantly exert catabolic effects on adult skeleton [7, 8]. Hyperthyroidism accelerates bone metabolism, leading to osteoporosis or osteopenia [7–9], affecting more bone resorption than formation [9]. Population studies indicate that hyperthyroidism is associated with an increased risk of fractures [10]. In some studies, even low-normal TSH values were associated with a high prevalence of vertebral fractures in women with postmenopausal osteoporosis or osteopenia, independently of thyroid hormones, age, and BMD [11].

In our study sclerostin concentrations were elevated during hyperthyroidism and decreased after restoration of an euthyroid state. Mean pretreatment sclerostin levels were even higher than reference values presented by Biomedica (43.7 ± 29.2 pmol/L versus 19.3 pmol/L, range from 10.9 to 28.7 pmol/L in serum) and by other authors, 33–37 pmol/L for healthy premenopausal women [3, 4].

Available data on the relationship between thyroid hormones and sclerostin are scarce. Our results are, however, convergent with recently published study by Tsourdi et al., performed on an animal model. In that research in hyperthyroid mice high sclerostin levels were observed, even when adjusted for bone mass. Moreover, sclerostin mRNA expression and the number of sclerostin-positive osteocytes were increased [12]. There are data suggesting that T_3_ inhibits Wnt signaling [13]. It cannot be ruled out that sclerostin is a mediator of thyroid hormone action on bone.

We hypothesized that decrease in sclerostin levels in patients recovering from hyperthyroidism might be mediated by PTH. In our study PTH levels during hyperthyroidism, that is, state associated with high FT_3_, were significantly lower than after treatment. A fall of PTH concentrations after triiodothyronine administration was demonstrated [14]. Decreased PTH concentrations in subjects with hyperthyroidism were also reported [15, 16], followed by an increase during treatment of hyperthyroidism [16]. There are many sets of data about inhibition of sclerostin expression by PTH both in vitro and in vivo. Activation of PTH receptor 1 expressed in osteocytes resulted in an increased bone remodelling with decreased osteoblast apoptosis and suppression of SOST expression [17]. In vivo, PTH administration resulted in lowering of sclerostin concentrations [18]. Decreased serum sclerostin levels were also shown in patients with high PTH concentration caused by primary hyperparathyroidism [19]; on the contrary in hypoparathyroidism sclerostin levels were high [20]. Inhibition of sclerostin by PTH is postulated to represent one of the mechanisms responsible for anabolic effect of PTH [21]. Nevertheless, it should be mentioned that in our study we failed to observe any direct correlation between sclerostin concentrations and PTH as well as between actual changes in sclerostin concentrations (i.e., Δsclerostin) versus ΔPTH. Therefore, an issue of precise mechanisms that mediate changes in sclerostin concentrations during treatment of thyrotoxicosis requires further study.

Analyzing other reasons for sclerostin decrease during treatment of hyperthyroidism one must also consider influence of bone markers. In our study there were elevated concentrations of bone markers (OC and CTX) before treatment while they decreased after normalization of thyroid function. High concentrations of bone markers, during hyperthyroidism, and their improvement after therapy had been demonstrated before [9, 16]. In our study, before starting therapy OC levels correlated with both FT_3_ and FT_4_, but after therapy we observed a conversion of that relationship to negative one. This might suggest that the above mentioned relationship might be more pertinent to pathological condition, that is, thyrotoxic state, rather than to euthyroidism. The positive correlation between sclerostin concentrations and CTX is an argument in support of hypothesis about relationship between elevated levels of sclerostin and increased bone metabolism. Similar relationship between sclerostin and CTX was shown in premenopausal [4] but not in postmenopausal women, in whom a negative correlation was found [22, 23]. However, we did not observe any direct relationship between sclerostin and concentrations of free thyroid hormones or TSH.

Another factor, potentially responsible for a decrease in sclerostin levels, might be related to an increase in physical activity during treatment of hyperthyroidism. There is evidence that mechanical loading is responsible for stimulation of bone formation, due to inhibition of sclerostin expression [1]. However, it is not clear whether this relationship is clinically significant. Armamento-Villareal et al. [24] did not find any change of sclerostin levels after one year of exercise, despite an increase of thigh muscle volume. We note, however, that no formal assessment of physical activity was performed in our study, while duration of treatment was relatively short (6–10 weeks), so a marked change in physical activity was unlikely to occur over such a short time span.

Moreover, we cannot exclude the influence of drugs used during therapy of hyperthyroidism on studied processes. All patients received a thyrostatic drug, thiamazole, and beta-blocker, propranolol. In literature there are no data on separate effect of such therapy on sclerostin or PTH levels; however in a follow-up study the reduction of fracture risk was observed in subjects that had received both radioactive iodine and thiamazole [25].

In turn, the data on the influence of beta-blockers, and propranolol in particular, on fracture risk are inconclusive, both in terms of risk reduction [26] and its increase [27].

Summing up, the precise pathomechanism responsible for a decrease of sclerostin concentrations during treatment of hyperthyroidism is still unclear and requires further study.

Limitations of our study included small number of treated patients and short time of observation. Nevertheless, all patients achieved biochemical euthyroidism, so these data are in our opinion suitable for a preliminary study, given that changes of sclerostin during treatment of an acute phase of thyrotoxicosis had not been described before.

We speculate that, in the context of high population prevalence of hyperthyroidism and its recurrent nature, our results might be clinically relevant in terms of potential benefits of prevention and treatment hyperthyroidism-induced osteoporosis with antibodies to sclerostin.

## Figures and Tables

**Table 1:** Descriptive statistics for demographic characteristics of the tested sample (*n* = 33) (*p* value of Wilcoxon's matched pairs test).

	Mean	SD	*p* value
Age [years]	48	15	—

Body mass, before [kg]	67	12	0.051
Body mass, after [kg]	69	11	

BMI value, before [kg/m^2^]	24.1	3.6	0.0024
BMI value, after [kg/m^2^]	24.6	3.6	

Height	1.67	0.8	—

**Table 2:** Comparison of distributions of selected parameters before and during treatment (*p* value of Wilcoxon's matched pairs test).

	Before treatment	During treatment	*p* value	Reference range
	(mean ± SD)	(mean ± SD)		
Sclerostin (pmol/L)	43.7 ± 29.2	28.1 ± 18.4	0.000001	10.9–28.7
Osteocalcin (ng/mL)	35.6 ± 22.0	27.0 ± 14.3	0.00004	15–46
CTX (ng/dL)	0.49 ± 0.35	0.35 ± 0.23	0.0016	0.13–0.71
Calcium (mmol/L)	2.47 ± 0.10	2.49 ± 0.38	0.061	2.2–2.55
PTH (pg/mL)	29.3 ± 14.9	39.8 ± 19.8	0.0005	15–65
FT_3_ (pg/mL)	10.16 ± 5.36	3.17 ± 1.03	0.000001	2.6–4.4
FT_4_ (pg/mL)	4.03 ± 2.33	1.10 ± 0.82	0.0006	0.93–1.7
TSH (*µ*IU/mL)	0.01 ± 0.01	1.27 ± 2.56	0.0001	0.27–4.2

**Table 3:** Spearman rank correlation coefficients before therapy. Analysis of association: significant correlations are marked with asterisks: ^*∗*^ *p* < 0.05,  ^*∗∗*^ *p* < 0.01. OC, osteocalcin; CTX, collagen type I cross-linked C-telopeptide.

Before treatment	BMI	AGE	TSH (mIU/L)	FT_3_ (pg/mL)	FT_4_ (ng/mL)	CTX (ng/dL)	OC (ng/mL)	PTH (pg/mL)
Sclerostin (pmol/L)	0.25	0.24	−0.22	−0.17	−0.09	0.41^*∗*^	0.16	−0.18
PTH (pg/mL)	0.14	0.17	0.15	−0.18	0.04	−0.01	0.06	
OC (ng/mL)	−0.22	−0.43^*∗*^	0.23	0.42^*∗*^	0.45^*∗*^	0.76^*∗∗*^		

**Table 4:** Spearman rank correlation coefficients after therapy. Analysis of association: significant correlations are marked with asterisks: ^*∗*^ *p* < 0.05, ^*∗∗*^ *p* < 0.01. OC, osteocalcin; CTX, collagen type I cross-linked C-telopeptide.

After treatment	BMI	AGE	TSH (mIU/L)	FT_3_ (pg/mL)	FT_4_ (ng/mL)	CTX (ng/dL)	OC (ng/mL)	PTH (pg/mL)
Sclerostin (pmol/L)	0.40^*∗*^	0.53^*∗∗*^	0.20	0.03	0.07	0.41^*∗*^	0.16	−0.18
PTH (pg/mL)	0.05	0.05	−0.06	−0.31	0.10	−0.01	0.06	
OC (ng/mL)	−0.06	−0.35	0.12	−0.45^*∗*^	−0.04	0.76^*∗∗*^		

## References

[1] Moester M. J. C., Papapoulos S. E., Löwik C. W. G. M., van Bezooijen R. L. (2010). Sclerostin: current knowledge and future perspectives. *Calcified Tissue International*.

[2] Baron R., Rawadi G. (2007). Targeting the Wnt/b-catenin pathway to regulate bone formation in the adults skeleton. *Endocrinology*.

[3] Ardawi M.-S. M., Al-Kadi H. A., Rouzi A. A., Qari M. H. (2011). Determinants of serum sclerostin in healthy pre- and postmenopausal women. *Journal of Bone and Mineral Research*.

[4] Amrein K., Amrein S., Drexler C. (2012). Sclerostin and its association with physical activity, age, gender, body composition, and bone mineral content in healthy adults. *Journal of Clinical Endocrinology and Metabolism*.

[5] Lewiecki E. M. (2014). Role of sclerostin in bone and cartilage and its potential as a therapeutic target in bone diseases. *Therapeutic Advances in Musculoskeletal Disease*.

[6] Skowrońska-Jóźwiak E., Krawczyk-Rusiecka K., Lewandowski K. C., Adamczewski Z., Lewiński A. (2012). Successful treatment of thyrotoxicosis is accompanied by a decrease in serum sclerostin levels. *Thyroid Research*.

[7] Gorka J., Taylor-Gjevre R. M., Arnason T. (2013). Metabolic and clinical consequences of hyperthyroidism on bone density. *International Journal of Endocrinology*.

[8] Svare A., Nilsen T. I. L., Bjøro T., Forsmo S., Schei B., Langhammer A. (2009). Hyperthyroid levels of TSH correlate with low bone mineral density: the HUNT 2 study. *European Journal of Endocrinology*.

[9] Nagasaka S., Sugimoto H., Nakamura T. (1997). Antithyroid therapy improves bony manifestations and bone metabolic markers in patients with Graves' thyrotoxicosis. *Clinical Endocrinology*.

[10] Vestergaard P., Mosekilde L. (2002). Fractures in patients with hyperthyroidism and hypothyroidism: a nationwide follow-up study in 16,249 patients. *Thyroid*.

[11] Mazziotti G., Porcelli T., Patelli I., Vescovi P. P., Giustina A. (2010). Serum TSH values and risk of vertebral fractures in euthyroid post-menopausal women with low bone mineral density. *Bone*.

[12] Tsourdi E., Rijntjes E., Köhrle J., Hofbauer L. C., Rauner M. (2015). Hyperthyroidism and hypothyroidism in male mice and their effects on bone mass, bone turnover, and the Wnt inhibitors sclerostin and dickkopf-1. *Endocrinology*.

[13] O'Shea P. J., Kim D. W., Logan J. G. (2012). Advanced bone formation in mice with a dominant-negative mutation in the thyroid hormone receptor *β* gene due to activation of Wnt/*β*-catenin protein signaling. *The Journal of Biological Chemistry*.

[14] Kobe N., Takamatsu J., Ito M., Sakane S., Ohsawa N. (1999). Acute and early effects of triiodothyronine administration on serum markers of bone and mineral metabolism. *Endocrine*.

[15] Bouillon R., De Moor P. (1974). Parathyroid function in patients with hyper- or hypothyroidism. *Journal of Clinical Endocrinology and Metabolism*.

[16] Pantazi H., Papapetrou P. D. (2000). Changes in parameters of bone and mineral metabolism during therapy for hyperthyroidism. *Journal of Clinical Endocrinology and Metabolism*.

[17] Saini V., Marengi D. A., Barry K. J. (2013). Parathyroid hormone (PTH)/PTH-related peptide type 1 receptor (PPR) signaling in osteocytes regulates anabolic and catabolic skeletal responses to PTH. *The Journal of Biological Chemistry*.

[18] Yu E. W., Kumbhani R., Siwila-Sackman E., Leder B. Z. (2011). Acute decline in serum sclerostin in response to PTH infusion in healthy men. *The Journal of Clinical Endocrinology & Metabolism*.

[19] Viapiana O., Fracassi E., Troplini S. (2013). Sclerostin and DKK1 in primary hyperparathyroidism. *Calcified Tissue International*.

[20] Costa A. G., Cremers S., Rubin M. R. (2011). Circulating sclerostin in disorders of parathyroid gland function. *Journal of Clinical Endocrinology and Metabolism*.

[21] Silva B. C., Costa A. G., Cusano N. E., Kousteni S., Bilezikian J. P. (2011). Catabolic and anabolic actions of parathyroid hormone on the skeleton. *Journal of Endocrinological Investigation*.

[22] Costa A. G., Walker M. D., Zhang C. A. (2013). Circulating sclerostin levels and markers of bone turnover in chinese-american and white women. *Journal of Clinical Endocrinology and Metabolism*.

[23] He J., Zhang H., Wang C. (2014). Associations of serum sclerostin and polymorphisms in the SOST gene with bone mineral density and markers of bone metabolism in postmenopausal chinese women. *Journal of Clinical Endocrinology and Metabolism*.

[24] Armamento-Villareal R., Aguirre L., Napoli N. (2014). Changes in thigh muscle volume predict bone mineral density response to lifestyle therapy in frail, obese older adults. *Osteoporosis International*.

[25] Vestergaard P., Rejnmark L., Weeke J., Mosekilde L. (2000). Fracture risk in patients treated for hyperthyroidism. *Thyroid*.

[26] Toulis K. A., Hemming K., Stergianos S., Nirantharakumar K., Bilezikian J. P. (2014). *β*-adrenergic receptor antagonists and fracture risk: a meta-analysis of selectivity, gender, and site-specific effects. *Osteoporosis International*.

[27] Choi H. J., Park C., Lee Y., Ha Y., Jang S., Shin C. S. (2015). Risk of fractures in subjects with antihypertensive medications: a nationwide claim study. *International Journal of Cardiology*.

